# Cardiovascular Toxicity With PD-1/PD-L1 Inhibitors in Cancer Patients: A Systematic Review and Meta-Analysis

**DOI:** 10.3389/fimmu.2022.908173

**Published:** 2022-07-08

**Authors:** Surui Liu, Wei Gao, Yan Ning, Xiaomeng Zou, Weike Zhang, Liangjie Zeng, Jie Liu

**Affiliations:** ^1^Department of Oncology, Central Hospital Affiliated to Shandong First Medical University, Jinan, China; ^2^Department of Pathology, Central Hospital Affiliated to Shandong First Medical University, Jinan, China; ^3^Department of Urology Surgery, Central Hospital Affiliated to Shandong First Medical University, Jinan, China; ^4^Department of Oncology, Jinan Central Hospital, Shandong University, Jinan, China

**Keywords:** PD-1/PD-L1 inhibitors, malignancies, cardiovascular toxicity, immunotherapy, trials

## Abstract

**Background:**

PD-1/PD-L1 inhibitors have significantly improved the outcomes of those patients with various malignancies. However, the incidence of adverse events also increased. This meta-analysis aims to systematically evaluate the risk of cardiovascular toxicity in patients treated with PD-1/PD-L1 inhibitors.

**Materials and methods:**

We searched PubMed, Embase, the Cochrane Library databases for all randomized controlled trials (RCTs) comparing all-grade and grade 3-5 cardiovascular toxicity of single-agent PD-1/PD-L1 inhibitors to placebo/chemotherapy, PD-1/PD-L1 inhibitors combined with chemotherapy to chemotherapy, or PD-1/PD-L1 inhibitors combined with CTLA-4 inhibitors to single-agent immune checkpoint inhibitors (ICIs) and pooled our data in a meta-analysis stratified by tumor types and PD-1 or PD-L1 inhibitors. The Mantel-Haenszel method calculated the odds ratio (OR) and its corresponding 95% confidence intervals (CIs).

**Results:**

A total of 50 trials were included in the analysis. Single-agent PD-1/PD-L1 inhibitors increased the risk of all-grade cardiotoxicity compared with placebo (OR=2.11, 95%CI 1.02-4.36, *P=*0.04). Compared with chemotherapy, patients receiving PD-1/PD-L1 inhibitors combined with chemotherapy had a significant higher risk of all-grade (OR=1.53, 95%CI 1.18-1.99, *P=*0.001) and grade 3-5 cardiotoxicity (OR=1.63, 95%CI 1.11-2.39, *P=*0.01) cardiotoxicity, especially patients with non-small cell lung cancer (NSCLC) [all-grade cardiotoxicity (OR=1.97, 95%CI 1.14-3.41, *P=*0.02) and grade 3-5 cardiotoxicity (OR=2.15, 95%CI 1.08-4.27, *P=*0.03)]. Subgroup analysis showed that PD-1 inhibitors combined with chemotherapy were associated with a higher risk of grade 3-5 cardiotoxicity (OR=2.08, 95%CI 1.18-3.66, *P=*0.01). Compared with placebo or chemotherapy, single-agent PD-1/PD-L1 inhibitors did not increase the risk of all-grade of myocarditis, arrhythmia and hypertension. However, PD-1/PD-L1 inhibitors combined with chemotherapy increased the risk of all-grade arrhythmia (OR=1.63, 95%CI 1.07-2.46, *P=*0.02) [PD-L1 inhibitor-containing treatment (OR=1.75, 95%CI 1.09-2.80, *P=*0.02)], and the risk of all-grade hypertension (OR=1.34, 95%CI 1.02-1.77, *P=*0.04) and grade 3-5 hypertension (OR=1.54, 95%CI 1.10-2.15, *P=*0.01).

**Conclusions:**

Our results suggest that single-agent PD-1/PD-L1 inhibitors increase the risk of all-grade cardiotoxicity, PD-1/PD-L1 inhibitors combined with chemotherapy increase the risk of all-grade and grade 3-5 cardiotoxicity, especially in those patients treated with PD-1 inhibitor-containing treatment and those with NSCLC. In addition, PD-1/PD-L1 inhibitors combined with chemotherapy increase the risk of arrhythmia and hypertension. Therefore, this evidence should be considered when assessing the benefits and risks of PD-1/PD-L1 inhibitors in treating malignancies.

**Systematic Review Registration:**

https://www.crd.york.ac.uk/prospero/, identifier CRD42022303115.

## Introduction

In recent years, immune checkpoint inhibitors (ICIs) have revolutionized the treatment landscape for numerous malignancies and improved outcomes for those patients (1). T cell activation requires at least two signals. The first signal is delivered *via* the T cell Receptor (TCR) after recognizing antigen bound to MHC-I or -II molecules. The second signal is also called the co-stimulatory signal, which provides positive and negative signals modulating T cell function (2, 3). Programmed death 1 (PD-1) is discovered on the surface of T cells and binds with programmed cell death 1 ligand 1 (PD-L1), widely expressed on tumor cells to elicit the inhibitory second signal for functional suppression of T-cell responses and tumor immune escape in several malignancies (4). Therefore, blockade of the PD-1/PD-L1 interaction enhances immune recognition and stimulation of T cells to attack tumor cells. Since 2014, various PD-1/PD-L1 inhibitors have been approved as the standard treatment for melanoma, lung cancer, urothelial cancer, cervical cancer, gastric cancer, gastroesophageal junction cancer, esophageal cancer, breast cancer and other solid tumors (5). However, treatments of such ICIs are commonly accompanied by immune-related adverse events (irAEs), which affect any organ, including skin, gastrointestinal, lung, liver, endocrine system, nervous system and cardiovascular systems (6, 7).

PD-1/PD-L1 inhibitors-related cardiovascular toxicity includes myocarditis, hypertension, heart failure, pericardial disease, prolonged QT interval, heart block, left ventricular insufficiency, decreased ejection fraction, ventricular arrhythmia, cardiogenic shock and cardiac arrest (8–10). Once myocarditis occurs, its fatality rate is about 39.7%-50% (11, 12). Therefore, cardiovascular toxicity caused by PD-1/PD-L1 inhibitors has become the research focus. Firstly, we need to know whether PD-1/PD-L1 inhibitors increase the risk of cardiovascular toxicity. Rubio-Infante et al. demonstrated that dual ICIs therapies seemed to provoke a higher rate of cardiac irAEs than monotherapies or ICIs plus chemotherapy (13). However, another previous meta-analysis showed that ICIs as single or combination regimens was not associated with an increased risk of cardiotoxicity (14). This shows that, to date, it is unclear whether treatment with PD-1/PD-L1 inhibitors will increase the risk of cardiovascular toxicity. Therefore, we systematically reviewed the RCTs to evaluate the risks of all-grade and grade 3-5 cardiovascular toxicity in patients treated with PD-1/PD-L1 inhibitors and get a deep insight into its prediction and management.

## Materials and methods

### Search Strategy and Selection Criteria

This meta-analysis was designed and performed in accordance with the Preferred Reporting Items for Systematic Reviews and Meta-analyses (PRISMA) reporting guidelines (15). The statement was registered at International Prospective Register of Systematic Reviews (number CRD42022303115).

We searched PubMed, Embase and the Cochrane Library databases from January 2010 to May 2022 for all randomized controlled trials (RCTs). Based on PICOS (participants, interventions, comparisons, outcomes, and study design) guidelines (16), the Medical Subject Headings (MeSH) terms and their entry terms were: “immune checkpoint inhibitors”, “PD-1 inhibitors”, “PD-L1 inhibitors”, “PD-1”, “PD-L1”, “CTLA-4”, “pembrolizumab”, “nivolumab”, “sintilimab”, “tislelizumab”, “toripalimab”, “cemiplimab”, “camrelizumab”, “atezolizumab”, “avelumab”, “durvalumab”, “ipilimumab”, “tremelimumab”, “neoplasms”, “drug-related side effects and adverse reactions”, “adverse reactions”, “randomized controlled trial”. The search was also restricted to articles published in English.

### Study Selection and Data Extraction

All RCTs related to PD-1/PD-L1 inhibitors or CTLA-4 inhibitors were phase III clinical trials. These RCTs included studies that compare single-agent PD-1/PD-L1 inhibitors with placebo/chemotherapy, PD-1/PD-L1 inhibitors plus chemotherapy with chemotherapy alone, or PD-1/PD-L1 inhibitors plus CTLA-4 inhibitors with single-agent ICIs. We only included studies if cardiovascular adverse events were reported, and the data were completely extractable. We excluded the trials published repeatedly. In addition, studies published as abstracts were excluded. Finally, those trials that reported no treatment-related cardiovascular toxicity were also excluded.

Data on cardiovascular toxicity was collected. Two researchers independently screened the titles and abstracts of identified publications, with any publication deemed potentially relevant by either researcher carried forward to full-text evaluation. If disagreement occurred, it was resolved by discussion with the corresponding author.

### Evaluation of Research Quality and Publication Bias

The Cochrane manual was used by two researchers to independently assess the bias risk of each included article (17), and the funnel plot and Egger’s test were used to assess publication bias (18). The included studies were evaluated for bias risk by RevMan5.4.1 Software, including random sequence generation and allocation concealment (selection bias), blinding of participants and personnel (performance bias), blindness to outcome assessment (detection bias), incomplete outcome data (attrition bias), selective outcome reporting (reporting bias), and other biases.

### Heterogeneity Assessment and Statistical Analysis

Review Manager (RevMan) version 5.4.1 was used to calculate the data. This analysis was stratified by tumor types and PD-1 or PD-L1 inhibitors. The Mantel-Haenszel method calculated OR and its corresponding 95% CIs. According to the recommendation of the Cochrane collaboration Network, Q statistics and I^2^ values were used to evaluate the heterogeneity among the included studies, which were divided into three grades: low, moderate and high according to the I^2^ value (< 25%, 25%-50%, > 50%). *P* < 0.05 or I^2^ > 50% was considered significant heterogeneity (19, 20). A random-effects model was used when significant heterogeneity existed, otherwise, a fixed-effects model was used. A funnel plot was used to evaluate publication bias. *P* values were two-tailed and statistical significance was set at 0.05.

## Results

**Figure 1:**
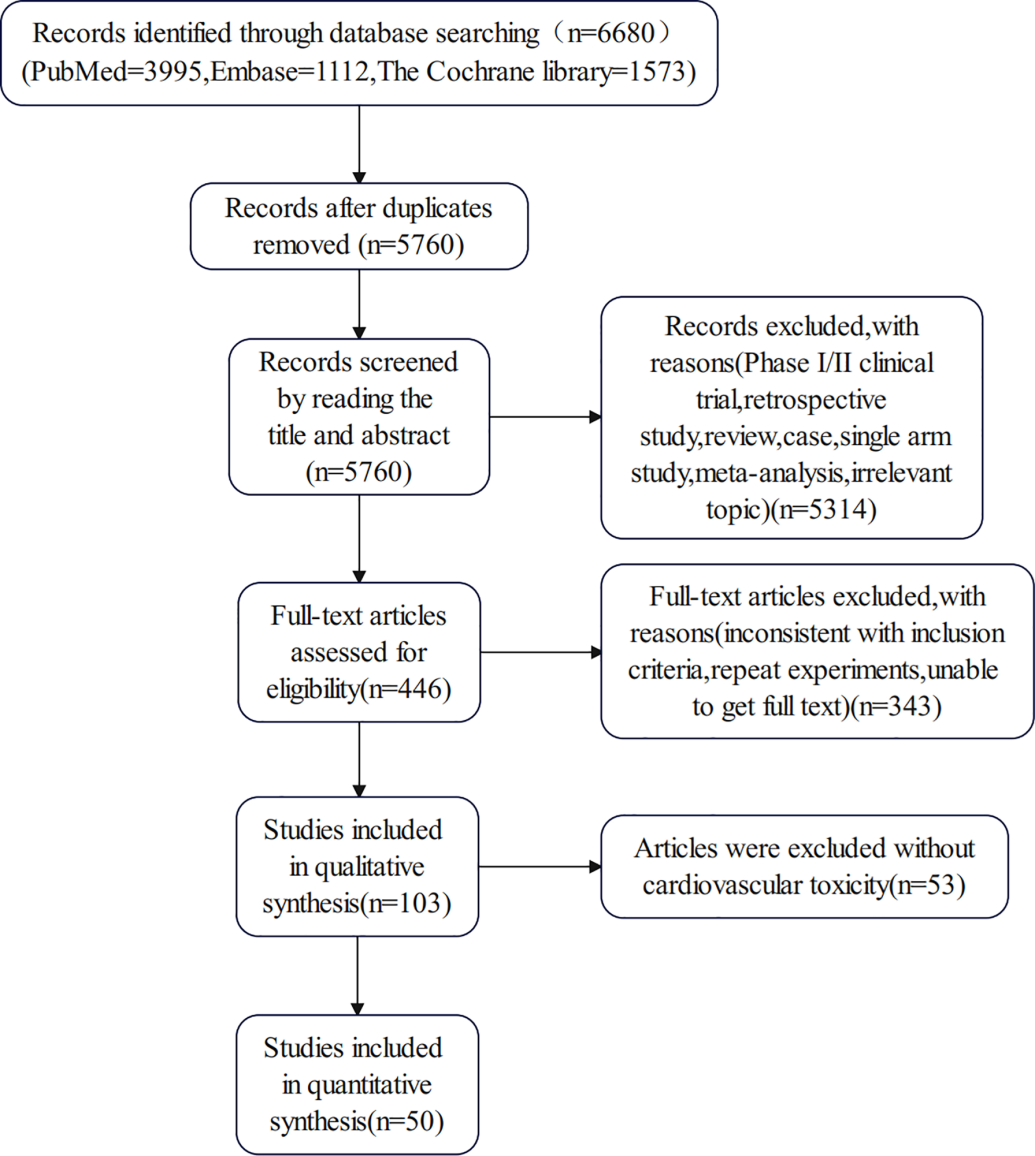
The flow diagram describing systematic search for RCTs.

**Figure 2:**
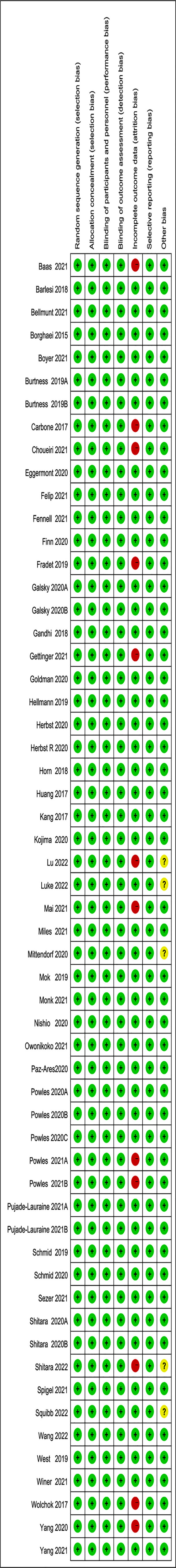
Risk bias diagram.

We retrieved 6680 potentially relevant trials from PubMed, Embase and the Cochrane Library databases. After removing duplicates and screening titles, abstracts, and full texts, 50 articles met our inclusion criteria and were included in the meta-analysis (21–70). There was a high-risk bias in 11 clinical trials, mainly due to incomplete reported data and other biases in 5 studies. The flow diagram of our search strategy and study selection is shown in **Figure 1**, and the quality assessment of included studies is shown in **Figure 2**. Publication bias is shown in the **Supplementary Figure**.

### Characteristics of Clinical Trials

The characteristics of 50 included clinical trials are shown in **Table 1**. 35 clinical trials included PD-1 inhibitors in the regimens, and 15 clinical trials included PD-L1 inhibitors. 8 clinical studies were carried out to compare PD-1/PD-L1 inhibitors with placebo, 18 studies about PD-1/PD-L1 inhibitors vs. chemotherapy, 21 studies about PD-1/PD-L1 inhibitors plus chemotherapy vs. chemotherapy alone, 4 studies about PD-1/PD-L1 inhibitors in combination with CTLA-4 inhibitors vs. chemotherapy, 5 studies about PD-1/PD-L1 inhibitors in combination with CTLA-4 inhibitors vs. PD-1/PD-L1 inhibitors alone.

**Table 1:** Characteristics of 50 studies included in analysis of cardiotoxicity and hypertension.

NO	Article	Clinical trial	Cancer type	Drug name	Treatment Regimen	Enrollment	All grade cardiotoxicity	Grade3-5 cardiotoxicity	All grade hypertension	Grade3-5hypertension
PD-1/PD-L1 VS Placebo										
1	54	MK-3475-054/1325-MG/KEYNOTE-54(NCT02362594)	Melanoma	Pembrolizumab(PD-1)	PembrolizumabVSPlacebo	1011	1	1	0	0
2	68	KEYNOTE-716(NCT02500121)	Melanoma	Pembrolizumab(PD-1)	PembrolizumabVSPlacebo	969	0	1	0	1
3	55	KEYNOTE-564(NCT03142334)	RCC	Pembrolizumab(PD-1)	PembrolizumabVSPlacebo	984	15	N/A	1	1
4	45	KEYNOTE-240(NCT02702401)	HCC	Pembrolizumab(PD-1)	PembrolizumabVSPlacebo	413	2	2	0	0
5	51	ONO-4538-12/ATTRACTION-2(NCT02267343)	GC	Nivolumab(PD-1)	NivolumabVSPlacebo	491	1	1	0	0
6	52	CONFIRM(NCT03063450)	MPM	Nivolumab(PD-1)	NivolumabVSPlacebo	332	10	2	0	0
7	62	IMpower010(NCT02486718)	NSCLC	Atezolizumab(PD-L1)	AtezolizumabVSBestSupportiveCare	990	2	0	0	0
8	56	IMvigor010(NCT02450331)	UC	Atezolizumab(PD-L1)	AtezolizumabVSPlacebo	809	1	1	0	0
PD-1/PD-L1 VS Chemotherapy										
1	22	KEYNOTE-361(NCT02853305)	UC	Pembrolizumab(PD-1)	PembrolizumabVSGemcitabine+Carboplatin	644	58	24	0	0
2	24	KEYNOTE-048(NCT02358031)	HNC	Pembrolizumab(PD-1)	PembrolizumabVSCetuximab+Platinum+5-Fluorouracil	587	0	0	8	8
3	25	KEYNOTE-062(NCT02494583)	GC	Pembrolizumab(PD-1)	PembrolizumabVSCisplatin+Fluorouracil/Capecitabine	498	1	1	0	0
4	36	KEYNOTE-010(NCT01905657)	NSCLC	Pembrolizumab(PD-1)	PembrolizumabVSDocetaxel	991	2	2	0	0
5	39	KEYNOTE-042(NCT02220894)	NSCLC	Pembrolizumab(PD-1)	PembrolizumabVSPlatinum-basedChemotherapy	1251	4	4	0	0
6	37	KEYNOTE-181(NCT02564263)	EC	Pembrolizumab(PD-1)	PembrolizumabVSPaclitaxel/Docetaxel/Irinotecan	610	1	1	0	0
7	38	KEYNOTE-119(NCT02555657)	BC	Pembrolizumab(PD-1)	PembrolizumabVSCapecitabine/Eeribulin/Gemcitabine/Vinorelbine	601	1	1	0	0
8	64	KEYNOTE-045(NCT02256436)	UC	Pembrolizumab(PD-1)	PembrolizumabVSPaclitaxel/Docetaxel/Vinflunine	521	31	7	0	0
9	66	CheckMate078(NCT02613507)	NSCLC	Nivolumab(PD-1)	NivolumabVSDocetaxel	493	N/A	8	0	0
10	61	CheckMate026(NCT02041533)	NSCLC	Nivolumab(PD-1)	NivolumabVSPlatinum-basedChemotherapy	530	2	2	0	0
11	40	CheckMate331(NCT02481830)	SCLC	Nivolumab(PD-1)	NivolumabVSTopotecan/Amrubicin	547	1	1	0	0
12	41	CheckMate057(NCT01673867)	NSCLC	Nivolumab	NivolumabVSDocetaxel	555	2	2	0	0
13	42	EMPOWER-Lung1(NCT03088540)	NSCLC	Cemiplimab(PD-1)	CemiplimabVSPlatinum-doubletChemotherapy	697	7	7	2	2
14	43	ESCORT(NCT03099382)	EC	Camrelizumab(PD-1)	CamrelizumabVSDocetaxel/Irinotecan	448	7	6	3	1
15	44	IMvigor130(NCT02807636)	UC	Atezolizumab(PD-L1)	AtezolizumabVSGemcitabine+Carboplatin	744	0	0	N/A	15
16	28	IMpower110(NCT02409342)	NSCLC	Atezolizumab(PD-L1)	AtezolizumabVSPlatinum-basedChemotherapy	549	5	5	14	5
17	57	JAVELINOvarian200(NCT02580058)	OC	Avelumab(PD-L1)	AvelumabVSPegylatedLiposomalDoxorubicin	364	5	3	4	2
18	46	JAVELINLung200(NCT02395172)	NSCLC	Avelumab(PD-L1)	AvelumabVSDocetaxel	758	3	3	5	3
19	50	DANUBE(NCT02516241)	UC	Durvalumab(PD-L1)	DurvalumabVSGemcitabine+Cisplatin/Carboplatin	686	5	5	0	0
PD-1/PD-L1+CTLA-4 VS Chemotherapy										
1	47	CheckMate743(NCT02899299)	MPM	Nivolumab(PD-1)	Nivolumab+IpilimumabVSPemetrexed+Cisplatin	584	5	5	0	0
2	49	CheckMate227(NCT02477826)	NSCLC	Nivolumab(PD-1)	Nivolumab+IpilimumabVSPlatinum-doubletChemotherapy	1146	2	2	0	0
3	70	CheckMate649(NCT02872116)	GEJC	Nivolumab(PD-1)	Nivolumab+IpilimumabVSOxaliplatin+Capecitabine/Leucovorin+Fluorouracil+Oxaliplatin	792	N/A	2	0	0
4	50	DANUBE(NCT02516241)	UC	Durvalumab(PD-L1)	Durvalumab+TremelimumabVSGemcitabine+Cisplatin/Carboplatin	653	1	1	0	0
PD-1/PD-L1+Chemotherapy VS Chemotherapy										
1	23	KEYNOTE-189(NCT02578680)	NSCLC	Pembrolizumab(PD-1)	Pembrolizumab+Pemetrexed+Platinum VS Pemetrexed+Platinum	607	5	5	0	0
2	26	KEYNOTE-407(NCT02775435)	NSCLC	Pembrolizumab(PD-1)	Pembrolizumab+Carboplatin+Paclitaxel/Nab-paclitaxel VS Carboplatin+Paclitaxel/ Nab-paclitaxel	559	2	2	0	0
3	22	KEYNOTE-361(NCT02853305)	UC	Pembrolizumab(PD-1)	Gemcitabine+CarboplatinVSGemcitabine+Carboplatin	691	69	28	0	0
4	33	KEYNOTE-522(NCT03036488)	BC	Pembrolizumab(PD-1)	Pembrolizumab+Epirubicin/Doxorubicin+Cyclophosphamide+CarboplatinandPaclitaxelVSEpirubicin/Doxorubicin+Cyclophosphamide+Carboplatin+PaclitaxelPembrolizumab+Platinum+	1170	3	2	0	0
5	24	KEYNOTE-048(NCT02358031)	HNC	Pembrolizumab(PD-1)	5-FluorouracilVSCetuximab+Platinum+5-Fluorouracil	563	1	1	12	12
6	25	KEYNOTE-062(NCT02494583)	GC	Pembrolizumab(PD-1)	Pembrolizumab+Cisplatin+Fluorouracil/CapecitabineVSCisplatin+Fluorouracil/Capecitabine	494	1	1	0	0
7	65	ORIENT-11(NCT03607539)	NSCLC	Sintilimab(PD-1)	Sintilimab+Pemetrexed+PlatinumVSPemetrexed+Platinum	397	N/A	4	0	0
8	67	ORIENT-15(NCT03748134)	EC	Sintilimab(PD-1)	Sintilimab+Paclitaxel/5-Fluorouracil+CisplatinVSPaclitaxel/5-Fluorouracil+Cisplatin	659	N/A	3	23	14
9	63	CAPTAIN-1st(NCT03707509)	NPC	Camrelizumab(PD-1)	Camrelizumab+Gemcitabine+CisplatinVSGemcitabine+Cisplatin	263	5	2	10	6
10	69	JUPITER-06(NCT03829969)	EC	Toripalimab(PD-1)	Toripalimab+Paclitaxel+PlatinumVSPaclitaxel+Platinum	514	12	2	2	0
11	27	NCT03581786	NPC	Toripalimab(PD-1)	Toripalimab+Gemcitabine+CisplatinVSGemcitabine+Cisplatin	289	2	1	N/A	12
12	29	IMpower133(NCT02763579)	SCLC	Atezolizumab(PD-L1)	Atezolizumab+Carboplatin+EtoposideVSCarboplatin+Etoposide	394	1	1	0	0
13	34	IMpower130(NCT02367781)	NSCLC	Atezolizumab(PD-L1)	Atezolizumab+Carboplatin+Nab-paclitaxelVSCarboplatin+Nab-paclitaxel	705	70	37	32	9
14	30	IMpower132(NCT02657434)	NSCLC	Atezolizumab(PD-L1)	Atezolizumab+Carboplatin/ Cisplatin+Pemetrexed VS Carboplatin/Cisplatin+Pemetrexed	565	2	2	0	0
15	31	IMpassion031(NCT03197935)	BC	Atezolizumab(PD-L1)	Atezolizumab+Nab-paclitaxelVSNab-paclitaxel	331	0	0	31	22
16	32	IMpassion131(NCT03125902)	BC	Atezolizumab(PD-L1)	Atezolizumab+PaclitaxelVSPaclitaxel	649	2	2	0	0
17	33	IMpassion130(NCT02425891)	BC	Atezolizumab(PD-L1)	Atezolizumab+Nab-paclitaxelVSNab-paclitaxel	890	40	22	48	15
18	44	IMvigor130(NCT02807636)	UC	Atezolizumab(PD-L1)	Atezolizumab+Gemcitabine+CarboplatinVSGemcitabine+Carboplatin	843	0	0	N/A	25
19	58	JAVELIN Ovarian 100(NCT02718417)	OC	Avelumab(PD-L1)	Avelumab+Paclitaxel+Carboplatin VS Paclitaxel+Carboplatin	991	45	6	49	15
20	57	JAVELIN Ovarian 200(NCT02580058)	OC	Avelumab(PD-L1)	Avelumab+Pegylated Liposomal DoxorubicinVS Pegylated Liposomal Doxorubicin	359	7	1	2	2
21	35	CASPIAN(NCT03043872)	SCLC	Durvalumab(PD-L1)	Durvalumab+Platinum+Etoposide VS Platinum+Etoposide	531	4	4	23	9
PD-1/PD-L1+CTLA-4 VS PD-1/PD-L1										
1	59	KEYNOTE-598(NCT03302234)	NSCLC	Pembrolizumab(PD-1)	Pembrolizumab+Ipilimumab VS Pembrolizumab	563	4	2	0	0
2	60	Lung- MAP S1400I(NCT02785952)	NSCLC	Nivolumab(PD-1)	Nivolumab+Ipilimumab VS Nivolumab	247	3	3	5	5
3	48	CheckMate 067(NCT01844505)	Melanoma	Nivolumab(PD-1)	Nivolumab+Ipilimumab VS Nivolumab	626	1	1	0	0
4	53	CheckMate451(NCT02538666)	SCLC	Nivolumab(PD-1)	Nivolumab+IpilimumabVSNivolumab	557	1	1	0	0
5	50	DANUBE(NCT02516241)	UC	Durvalumab(PD-L1)	Durvalumab+TremelimumabVSDurvalumab	685	4	4	0	0

RCC: Renal Cell Carcinoma; UC: Urothelial Carcinoma; HCC: Hepatocellular Carcinoma; GC: Gastric Cancer ;GJEC: Gastro-oesophageal Junction Cancer; MPM: Malignant Pleural Mesothelioma; NSCLC: Non-Small Cell Lung Cancer; SCLC: Small Cell Lung Cancer; HNC: Head and Neck Cancer; EC: Esophageal Cancer; BC: Breast Cancer; OC: Ovarian Cancer; NPC: Nasopharyngeal Carcinoma; N/A: No Available.

### Risk of Cardiotoxicity

**Figure 3:**
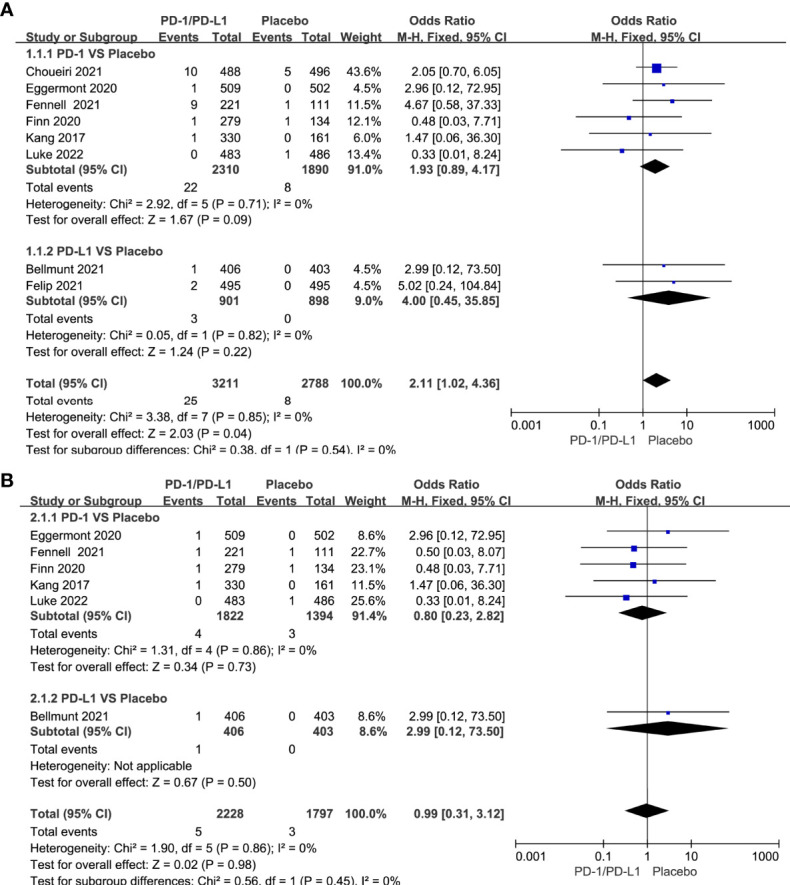
Forest plots of the risk of all-grade and grade 3-5 cardiotoxicity calculated by the fixed effect model. **(A)** The risk of all-grade cardiotoxicity in PD-1/PD-L1 inhibitors VS Placebo group. **(B)** The risk of grade 3-5 cardiotoxicity in PD-1/PD-L1 inhibitors VS Placebo group.

The incidence of cardiovascular toxicity in different treatment groups is shown in **Table 2**. Compared with placebo, single-agent PD-1/PD-L1 inhibitors had a higher risk of all-grade cardiotoxicity (OR=2.11, 95%CI 1.02-4.36, *P=*0.04) (**Figure 3A**), and no significant difference in the risk of grade 3-5 cardiotoxicity was found (OR=0.99, 95%CI 0.31-3.12, *P=*0.98) (**Figure 3B**).

**Table 2:** The incidence of cardiovascular toxicity in different treatment groups. .

AE	PD-1/PD-L1/N (%)		PD-1/PD-L1+Chemotherapy/N (%)		PD-1/PD-L1+CTLA-4/N (%)		Placebo/N (%)		Chemotherapy/N (%)	
	All-grade	Grade3-5	All-grade	Grade3-5	All-grade	Grade3-5	All-grade	Grade3-5	All-grade	Grade3-5
Myocarditis	13 (0.13)	7 (0.07)	9 (0.14)	7 (0.11)	7 (0.27)	7 (0.27)	1 (0.04)	1 (0.04)	1 (0.01)	1 (0.01)
Myocardial infarction	11 (0.11)	8 (0.08)	7 (0.11)	7 (0.11)	0	0	1 (0.04)	0	11 (0.10)	11 (0.10)
Pericardial effusion	8 (0.08)	8 (0.08)	8 (0.12)	6 (0.09)	0	0	0	0	1 (0.01)	0
Arrhythmia	17 (0.17)	11 (0.11)	77 (1.17)	25 (0.38)	0	0	3 (0.11)	1 (0.04)	29 (0.27)	13 (0.12)
Heart failure	11 (0.11)	9 (0.09)	5 (0.08)	6 (0.09)	2 (0.08)	4 (0.15)	1 (0.04)	0	14 (0.13)	11 (0.10)
Cardiac arrest	6 (0.06)	6 (0.06)	10 (0.15)	10 (0.15)	1 (0.04)	1 (0.04)	0	0	5 (0.05)	5 (0.05)
Hypertension	22 (0.21)	18 (0.17)	145 (2.20)	96 (1.46)	4 (0.15)	4 (0.15)	0	0	99 (0.91)	61 (0.56)

10291 patients in PD-1/PD-L1 inhibitors monotherapy, 6584 patients in PD-1/PD-L1 inhibitors + Chemotherapy, 2616 patients in PD-1/PD-L1+CTLA-4 inhibitors, 2788 patients in Placebo and 10887 patients in Chemotherapy.

**Figure 4:**
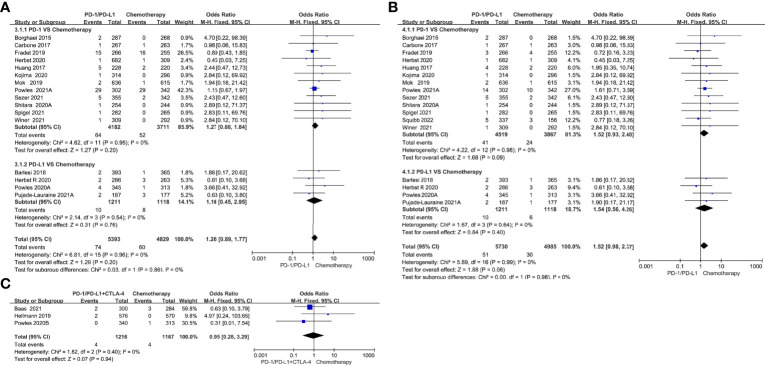
Forest plots of the risk of all-grade and grade 3-5 cardiotoxicity calculated by the fixed effect model. **(A)** The risk of all-grade cardiotoxicity in PD-1/PD-L1 inhibitors VS Chemotherapy group. **(B)** The risk of grade 3-5 cardiotoxicity in PD-1/PD-L1 inhibitors VS Chemotherapy group. **(C)** The risk of all-grade cardiotoxicity in PD-1/PD-L1+CTLA-4 inhibitors VS Chemotherapy group.

Compared with chemotherapy, single-agent PD-1/PD-L1 inhibitors did not increase the risk of all-grade cardiotoxicity (OR=1.26, 95%CI 0.89-1.77, *P=*0.20) and grade 3-5 cardiotoxicity (OR=1.52, 95%CI 0.98-2.37, *P=*0.06) (**Figures 4A, B**). Tumor type-stratified analyses showed no statistically significant differences in either all-grade or grade 3-5 cardiotoxicity among different tumor types (**Supplementary Figure 1**). Compared with chemotherapy, PD-1/PD-L1 inhibitors combined with CTLA-4 inhibitors did not increase the risk of all-grade of cardiotoxicity (OR=0.95, 95%CI 0.28-3.29, *P=*0.94) (**Figure 4C**). The corresponding funnel plots showed no obvious publication bias (**Supplementary Figures 2A–E**).

**Figure 5:**
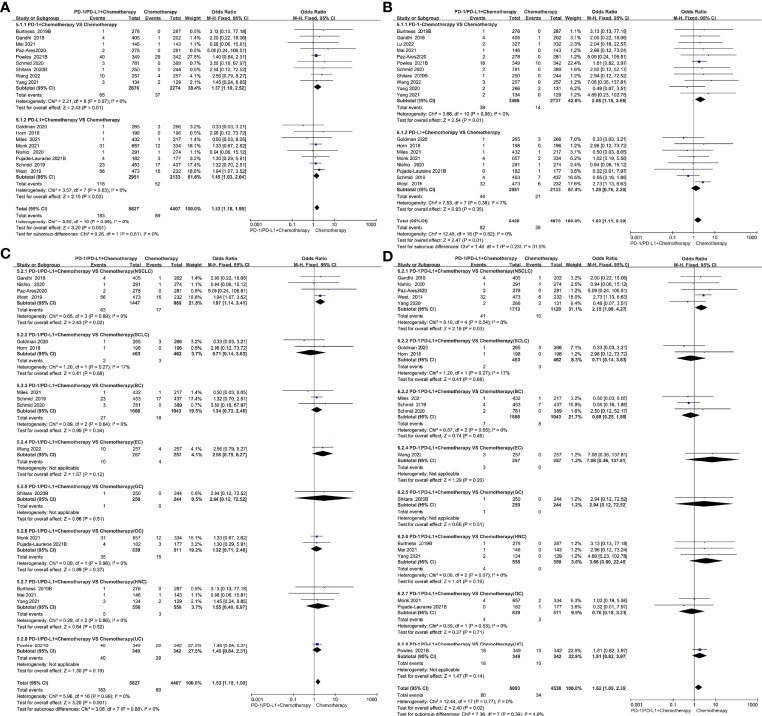
Forest plots of the risk of all-grade and grade 3-5 cardiotoxicity calculated by the fixed effect model. **(A)** The risk of all-grade cardiotoxicity in PD-1/PD-L1 inhibitors + Chemotherapy VS Chemotherapy group. **(B)** The risk of grade 3-5 cardiotoxicity in PD-1/PD-L1 inhibitors + Chemotherapy VS Chemotherapy group. **(C)** The risk of all-grade cardiotoxicity in PD-1/PD-L1 inhibitors + Chemotherapy VS Chemotherapy group (subgroup analysis was performed based on tumor types). **(D)** The risk of grade 3-5 cardiotoxicity in PD-1/PD-L1 inhibitors + Chemotherapy VS Chemotherapy group (subgroup analysis was performed based on tumor types).

Compared with chemotherapy, PD-1/PD-L1 inhibitors combined with chemotherapy increased the risk of all-grade cardiotoxicity (OR=1.53, 95%CI 1.18-1.99, *P=*0.001) and grade 3-5 cardiotoxicity (OR=1.63, 95%CI 1.11-2.39, *P=*0.01) (**Figures 5A, B**). Subgroup analysis suggested that PD-1 inhibitors combined with chemotherapy were associated with a higher incidence risk of grade 3-5 cardiotoxicity (OR=2.08, 95%CI 1.18-3.66, *P=*0.01) (**Figure 5B**); however, it was not observed in the patients undergoing PD-L1 inhibitors combined with chemotherapy. Tumor type-stratified analyses showed an increased risk of all-grade cardiotoxicity (OR=1.97, 95%CI 1.14-3.41, *P=*0.02) and grade 3-5 cardiotoxicity (OR=2.15, 95%CI 1.08-4.27, *P=*0.03) (**Figures 5C, D**) in patients with non-small cell lung cancer undergoing PD-1/PD-L1 inhibitors combined with chemotherapy. No statistically significant difference was observed in breast cancer, uroepithelial cancer, small cell lung cancer and other cancers. The corresponding funnel plots showed no obvious publication bias (**Supplementary Figures 3A–D**).

**Figure 6:**
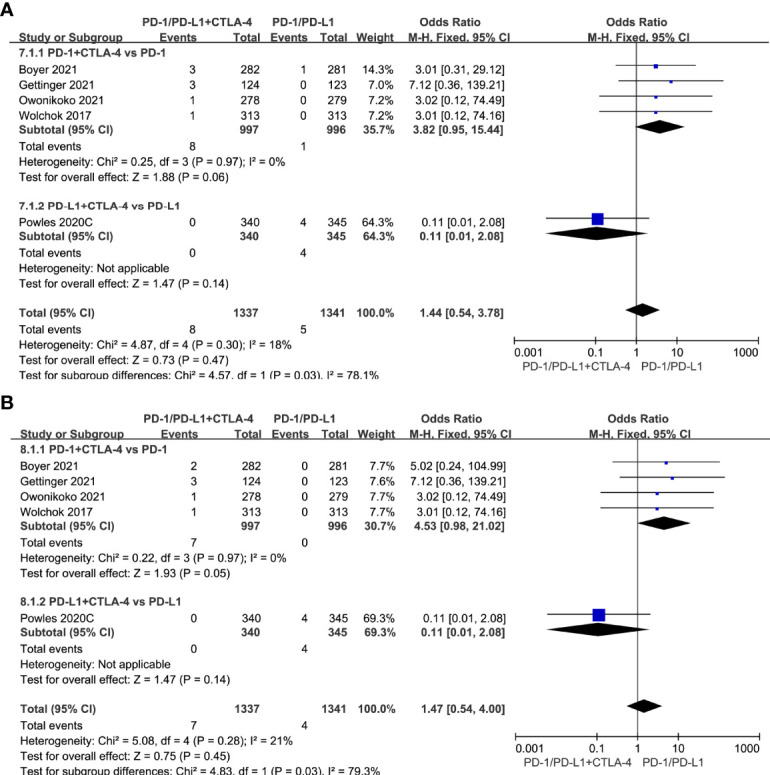
Forest plots of the risk of all-grade and grade 3-5 cardiotoxicity calculated by the fixed effect model. **(A)** The risk of all-grade cardiotoxicity in PD-1/PD-L1 inhibitors +CTLA-4 inhibitors VS PD-1/PD-L1 inhibitors group. **(B)** The risk of grade 3-5 cardiotoxicity in PD-1/PD-L1 inhibitors +CTLA-4 inhibitors VS PD-1/PD-L1 inhibitors group.

Compared with single-agent PD-1/PD-L1 inhibitors, PD-1/PD-L1 inhibitors in combination with CTLA-4 inhibitors did not increase the risk of all-grade cardiotoxicity (OR=1.44, 95%CI 0.54-3.78, *P=*0.47) and grade 3-5 cardiotoxicity (OR=1.47, 95%CI 0.54-4.00, *P=*0.45) (**Figures 6A, B**).

### Risk of Myocarditis, Arrhythmia and Hypertension

All results are displayed in **Table 3**. Compared with placebo, single-agent PD-1/PD-L1 inhibitors did not increase the risk of all-grade myocarditis (OR=2.01, 95%CI 0.50-8.04, *P=*0.33) and arrhythmia (OR=1.83, 95%CI 0.47-7.18, *P=*0.39). We could not conduct statistical analyses because of the low incidence of grade 3-5 myocarditis and arrhythmias in the included studies. Compared with chemotherapy, single-agent PD-1/PD-L1 inhibitors did not increase the risk of all-grade and grade 3-5 myocarditis, arrhythmia and hypertension. Meanwhile, we did not find that PD-1/PD-L1 inhibitors with chemotherapy increased the risk of all grade (OR=2.79, 95%CI 0.83-9.37, *P=*0.10) and grade 3-5 (OR=2.34, 95%CI 0.68-8.06, *P=*0.18) myocarditis compared with chemotherapy. Whereas the patients treated with PD-1/PD-L1 inhibitors combined with chemotherapy had a significantly higher risk of all-grade arrhythmia (OR=1.63, 95%CI 1.07-2.46, *P=*0.02), especially those with PD-L1 inhibitors combined with chemotherapy (OR=1.75, 95%CI 1.09-2.80, *P=*0.02). The risk of grade 3-5 arrhythmia in different treatment groups was not statistically significant. Compared with chemotherapy, PD-1/PD-L1 inhibitors combined with chemotherapy increased the risk of all-grade hypertension (OR=1.34, 95%CI 1.02-1.77, *P=*0.04) and grade 3-5 hypertension (OR=1.54, 95%CI 1.10-2.15, *P=*0.01). Subgroup analysis suggested that PD-1 inhibitors combined with chemotherapy were associated with a higher incidence risk of all-grade (OR=2.17, 95%CI 1.17-4.00, *P=*0.01) and grade 3-5 hypertension (OR=2.01, 95%CI 1.08-3.73, *P=*0.03).

**Table 3:** The risk of all-grade and grade 3-5 myocarditis, arrhythmia and hypertension.

**Treatment Regimen**			PD-1/PD-L1 VS Placebo	PD-1/PD-L1 VS Chemotherapy	PD-1/PD-L1 +Chemotherapy VS Chemotherapy	PD-1 +Chemotherapy VS Chemotherapy	PD-L1 +Chemotherapy VS Chemotherapy
**Hypertension**	Grade 3-5	*P* value	N/A	0.53	**0.01**	**0.03**	0.13
		95%CI		0.43-1.54	1.10-2.15	1.08-3.73	0.92-2.04
		OR		0.81	1.54	2.01	1.37
	All grade	*P* value	N/A	0.86	**0.04**	**0.01**	0.29
		95%CI		0.57-1.98	1.02-1.77	1.17-4.00	0.87-1.61
		OR		1.06	1.34	2.17	1.18
**Arrhythmia**	Grade 3-5	*P* value		0.06	0.23	0.51	0.32
		95%CI	N/A	0.94-7.35	0.76-3.13	0.45-4.99	0.65-3.77
		OR		2.63	1.54	1.50	1.56
	All grade	*P* value	0.39	0.10	**0.02**	0.63	**0.02**
		95%CI	0.47-7.18	0.84-6.08	1.07-2.46	0.52-3.01	1.09-2.80
		OR	1.83	2.27	1.63	1.24	1.75
**Myocarditis**	Grade 3-5	*P* value		0.08	0.18	0.14	0.97
		95%CI	N/A	0.87-11.49	0.68-8.06	0.70-11.95	0.06-15.12
		OR		3.16	2.34	2.88	0.94
	All grade	*P* value	0.33	0.06	0.10	0.08	0.97
		95%CI	0.50-8.04	0.97-12.49	0.83-9.37	0.87-14.15	0.06-15.12
		OR	2.01	3.48	2.79	3.51	0.94

N/A, No Available.

## Discussion

The meta-analysis showed that the risk of all-grade cardiotoxicity in the patients undergoing single-agent PD-1/PD-L1 inhibitors was significantly higher than those with placebo. However, the subgroup analysis showed no significant difference in both PD-1 and PD-L1 inhibitors subgroups in spite of the tendency towards the increased risk. This result may be due to inadequate statistical power owing to the insufficient sample size. The risk of grade 3-5 cardiotoxicity was not statistically significant. Compared with chemotherapy, PD-1/PD-L1 inhibitors monotherapy did not increase the risk of all-grade and grade 3-5 cardiotoxicity. This may be due to different tumor types included in this study, and anthracyclines, fluorouracil, paclitaxel and other chemotherapeutic drugs were included in the meta-analysis, which could cause cardiotoxicity (24, 25, 37, 57, 64). Therefore, we believe that the safety of PD-1/PD-L1 inhibitors monotherapy is acceptable. However, patients using PD-1/PD-L1 inhibitors still need to regularly monitor cardiac function in a clinic, such as cardiac troponin, electrocardiogram (ECG), cardiac ultrasound, etc. Physicians should closely observe the clinical manifestations of these patients.

As described above, PD-1/PD-L1 inhibitors monotherapy increases the risk of cardiovascular toxicity, and chemotherapy could also lead to cardiovascular toxicity. Would PD-1/PD-L1 inhibitors combined with chemotherapy cause more severe cardiotoxicity? So far, the mechanism of ICIs-induced cardiovascular toxicity is unclear, and there are several widely held ideas about it: 1. PD-L1 could be expressed in normal cardiomyocytes or endothelial cells. When ICIs blocked the PD-1 and PD-L1 interaction, myocarditis, vasculitis, atherosclerosis, arrhythmia and pericardial diseases occurred (71); 2. Both cardiomyocytes and tumor cells expressed antigens recognized by T cells; When ICIs activate T cells and attack the tumor, it also causes autoimmune myocarditis (72); 3. Under the condition of inflammation, the upregulation of PD-L1 in the myocardium might be a cytokine-mediated cardioprotective mechanism, which was very important for limiting immune-mediated cardiac injury. The anti-PD-1 monoclonal antibody could inhibit the up-regulation of PD-L1 expression during cardiac injury and further aggravate cardiac inflammation (73). The mechanism of cardiovascular toxicity induced by chemotherapeutic drugs was different from that of PD-1/PD-L1 inhibitors. Anthracyclines could result in mitochondrial damage in cardiomyocytes and apoptosis through several distinct signaling pathways (74). Fluorouracil is a powerful inhibitor of the tricarboxylic acid cycle, interfering with cardiomyocyte energy metabolism and causing cardiomyocyte necrosis (75). Taxanes might cause myocardial injury by inhibiting tubulin activity and blocking cell division (76). Theoretically, PD-1/PD-L1 inhibitors combined with chemotherapy might increase the risk of cardiovascular toxicity. Rohit Bishnoi et al. conducted a retrospective analysis based on the Surveillance, Epidemiology, and End Results Program (SEER) database, including newly diagnosed metastatic non-small cell lung cancer (NSCLC) patients aged ≥ 65 years old from 2013 to 2015. They were divided into two groups: 675 patients received ICIs combined with traditional chemotherapy and 5730 patients received traditional chemotherapy. The primary endpoint was the hazards of new cardiovascular toxicity. The results showed that the hazard ratio for all cardiovascular toxicity was 0.81 (95% CI: 0.72− 0.91, *P=*0.0003) in patients who received ICIs with chemotherapy, and subgroup analysis showed a significant decrease in the risk of heart failure, arrhythmia and cardiogenic shock in patients who received ICIs with chemotherapy, but there was no significant difference in the risk of cardiomyopathy, pericarditis and myocarditis (77). However, in this study, PD-1/PD-L1 inhibitors combined with chemotherapy showed a significant increase in the risk of all-grade and grade 3-5 cardiotoxicity compared with the chemotherapy. This difference might be because the studies included in the meta-analysis were prospective and most studies excluded the patients older than age 75 years and with some complications. In contrast, another study was a retrospective analysis, and the study included the patients older than age 65 years, having more complications or cardiovascular diseases, using more drugs. All of these factors might have a certain impact on the results.

The two factors were stratified in this study to determine the effects of different tumor types and PD-1/PD-L1 inhibitors on cardiovascular toxicity. The results showed that PD-1/PD-L1 inhibitors combined with chemotherapy increased all-grade and grade 3-5 cardiotoxicity, especially in those patients with NSCLC. However, no difference was observed in other cancers such as small cell lung cancer, breast cancer, etc. Firstly, the patient population with NSCLC was often elderly with multiple comorbid conditions (smoking history, hypertension, dyslipidemia, obesity) that increased the risk of heart disease (78). Secondly, different chemotherapy regimen was used for different cancer types. In NSCLC, the chemotherapy regimen of pemetrexed combined with cisplatin/carboplatin or paclitaxel combined with cisplatin/carboplatin has a relatively low risk of cardiotoxicity (0.4-1.5%) (23, 65). In breast cancer, the chemotherapy regimen contains anthracycline, and in head and neck cancer and gastric cancer fluorouracil is contained, inducing a relatively higher (0.7-3.8%) risk of chemotherapy-induced cardiotoxicity (27, 33). Several studies showed that PD-1 inhibitors had higher incidences of adverse events such as rash, colitis, liver damage, hypothyroidism and interstitial pneumonia than PD-L1 inhibitors (79, 80), so was this also the case for the incidence of cardiotoxicity? The results showed that PD-1 inhibitors combined with chemotherapy significantly increased grade 3-5 cardiotoxicity risk compared with the chemotherapy. However, PD-L1 combined with chemotherapy did not show the above results. Herein, we compared the PD-1/PD-L1 inhibitors combined with the CTLA-4 inhibitors with PD-1/PD-L1 inhibitors monotherapy, and found that there was no statistical difference in the risk of cardiotoxicity between the PD-1/PD-L1 inhibitors combined with the CTLA-4 inhibitors group and PD-1/PD-L1 inhibitors monotherapy, which was consistent with the previous report (81).

Cardiovascular toxicity of PD-1/PD-L1 inhibitors includes hypertension, myocarditis, arrhythmia, heart failure, myocardial infarction, pericardial effusion and cardiac arrest (8–10). We performed statistics on several cardiovascular toxicities included in the study, and the results showed that the incidence of all-grade myocarditis in the patients undergoing PD-1/PD-L1 inhibitors monotherapy was 0.13%, myocardial infarction 0.11%, pericardial effusion 0.08%, arrhythmia 0.17%, heart failure 0.11%, cardiac arrest 0.06% and hypertension 0.21%. The incidences of all-grade myocarditis in the patients receiving PD-1/PD-L1 inhibitors combined with chemotherapy were 0.14%, myocardial infarction 0.11%, pericardial effusion 0.12%, arrhythmia 1.17%, heart failure 0.08%, cardiac arrest 0.15% and hypertension 2.20%. Arrhythmia and hypertension are high incidences of cardiovascular toxicity. Although the incidence of immune-related myocarditis is only 0.41%-1.33%, its fatality rate is about 39.7%-50% (11, 12). Therefore, we compared the risk of myocarditis, arrhythmia and hypertension in this analysis.

To explore the effect of PD-1/PD-L1 inhibitors combination or monotherapy on myocarditis, we conducted a meta-analysis of 16 studies. Our results showed that compared with the placebo, single-agent PD-1/PD-L1 inhibitors did not increase the risk of myocarditis. Compared with chemotherapy, neither the PD-1/PD-L1 monotherapy nor the combination treatments increased the risk of all-grade and grade 3-5 myocarditis. To understand the manifestation and clinical course of immune-associated myocarditis, Mahmood SS et al. retrospectively analyzed 140 patients treated with ICIs. The results showed that the median time of onset of myocarditis was 34 days after the start of immunotherapy (interquartile range 21 to 75), 29% of the patients were female, and patients with diabetes, sleep apnea syndrome and a high body mass index were more likely to develop myocarditis (82). Patients with melanoma, NSCLC, heart disease or potential autoimmune diseases also face a higher risk of myocarditis (83). However, due to the limitations of the data, we could not discuss them separately. We found no statistically significance in the risk of myocarditis among the treatment groups, possibly because the study we included was prospective and related to the low incidence of myocarditis. However, for clinicians, myocarditis is a fatal adverse event, and people with risk factors should be highly vigilant against the occurrence of myocarditis in clinical applications.

The mechanism of arrhythmia caused by ICIs is still elusive. Wu et al. found that the release of proinflammatory cytokines, abnormal calcium homeostasis, direct myocardial injury, and increased vagus nerve and adrenergic tension would interfere with the heart conduction system (84). Various arrhythmia was reported in immunotherapy patients, including atrial fibrillation, atrioventricular block, tachycardia, bradycardia, etc. (78). We analyzed patients with arrhythmia and showed that PD-1/PD-L1 inhibitors monotherapy did not increase the risk of arrhythmia compared with chemotherapy or placebo. Compared with chemotherapy, PD-1/PD-L1 inhibitors combined with chemotherapy increased the risk of all-grade arrhythmia. This trend was observed in the PD-L1 inhibitors combined with the chemotherapy group. Mirabel M et al. conducted a statistical analysis of the World Health Organization database of Individual Case Safety Report (ICSR) to evaluate the risk of ventricular arrhythmia treated with ICIs. A similar result was obtained that avelumab was associated with fatal arrhythmia (85). However, the mechanism of arrhythmia mediated by PD-L1 inhibitors was not clear, which might be related to the diversified expression of PD-L1 in many non-hematopoietic tissues, including the heart, pancreas, placenta, vascular endothelium, liver, lung and skin, and it was usually up-regulated with activation (86). As few trials were related to avelumab, we did not analyze it separately, which can be further confirmed in the subsequent studies.

Hypertension is common cardiovascular toxicity (9). We reviewed the relevant literature and found that no study reported the effect of ICIs therapy on the risk of hypertension, so we counted the incidence of hypertension in the included study. The results showed that the incidence of all-grade hypertension was 0.21% in the PD-1/PD-L1 inhibitors monotherapy group and 0.91% in the chemotherapy group, but increased to 2.20% in the PD-1/PD-L1 inhibitors combined with chemotherapy group. We conducted a meta-analysis of the treatment group, there was no significant difference in the risk of all-grade and grade 3-5 hypertension in the PD-1/PD-L1 inhibitors monotherapy group compared with chemotherapy. Meanwhile, the risk of all-grade and grade 3-5 hypertension in the PD-1/PD-L1 inhibitors combined with chemotherapy group were increased. This trend was more obvious in the PD-1 inhibitors combined with chemotherapy. PD-1/PD-L1 inhibitors combined with chemotherapy increased the risk of hypertension, but the mechanism is not clear and further study is needed. Therefore, we need to regularly monitor the blood pressure of patients treated with PD-1/PD-L1 inhibitors combined with chemotherapy. In the PD-1/PD-L1 inhibitors combined with CTLA-4 inhibitors group, the incidence of hypertension is lower and the data is less, so we cannot analyze this part of the patients.

There are several limitations in our study. Firstly, we analyzed data from clinical trials, so we cannot rule out confounding factors, such as previous treatment of patients. Secondly, some studies have shown that cardiovascular toxicity is more common in male patients (12, 87), however, we could not conduct a gender subgroup analysis because of the limitations of the data. Thirdly, some clinical trials presented data only for high or severe cardiovascular toxicity, so we may underestimate the risk of cardiovascular toxicity.

## Conclusion

To sum up, single-agent PD-1/PD-L1 inhibitors increase the risk of all-grade cardiotoxicity, PD-1/PD-L1 inhibitors combined with chemotherapy increase all-grade and grade 3-5 cardiotoxicity, especially in those patients treated with PD-1 inhibitors and those with NSCLC. Moreover, PD-1/PD-L1 inhibitors combined with chemotherapy increase the risk of arrhythmia and hypertension. Therefore, this evidence should be considered when assessing the benefits and risks of PD-1/PD-L1 inhibitors in treating malignancies. Clinicians should pay more attention to regularly monitoring the cardiac function of these patients to reduce the risk of cardiovascular toxicity, improve the quality of life and prolong the survival time of patients.

## Data Availability Statement

The original contributions presented in the study are included in the article/**Supplementary Material**. Further inquiries can be directed to the corresponding author.

## Author Contributions

Data collection and analysis: SL, WG, YN, XZ, and WZ. Data interpretation: SL, WG, and YN. Quality Evaluation: SL, WG, YN, and LZ. Writing - first draft: SL. Writing - Review and Editing: JL. All authors contributed to the article and approved the submitted version.

## Funding

This work was supported by the Natural Science Foundation of Shandong Province (ZR2020MH210, ZR2020QH179), and the Jinan Medical and Health Science and Technology Development Project (grant no. 201907118).

## Conflict of Interest

The authors declare that the research was conducted in the absence of any commercial or financial relationships that could be construed as a potential conflict of interest.

## Publisher’s Note

All claims expressed in this article are solely those of the authors and do not necessarily represent those of their affiliated organizations, or those of the publisher, the editors and the reviewers. Any product that may be evaluated in this article, or claim that may be made by its manufacturer, is not guaranteed or endorsed by the publisher.
