# Supplementary material for: Cardiovascular Toxicity With PD-1/PD-L1 Inhibitors in Cancer Patients: A Systematic Review and Meta-Analysis
Source: Front Immunol. 2022 Jul 8;13:908173. doi: 10.3389/fimmu.2022.908173 (PMC9307961; doi:10.3389/fimmu.2022.908173)
Supplement: Supplementary file 1 [file DataSheet_1.docx]

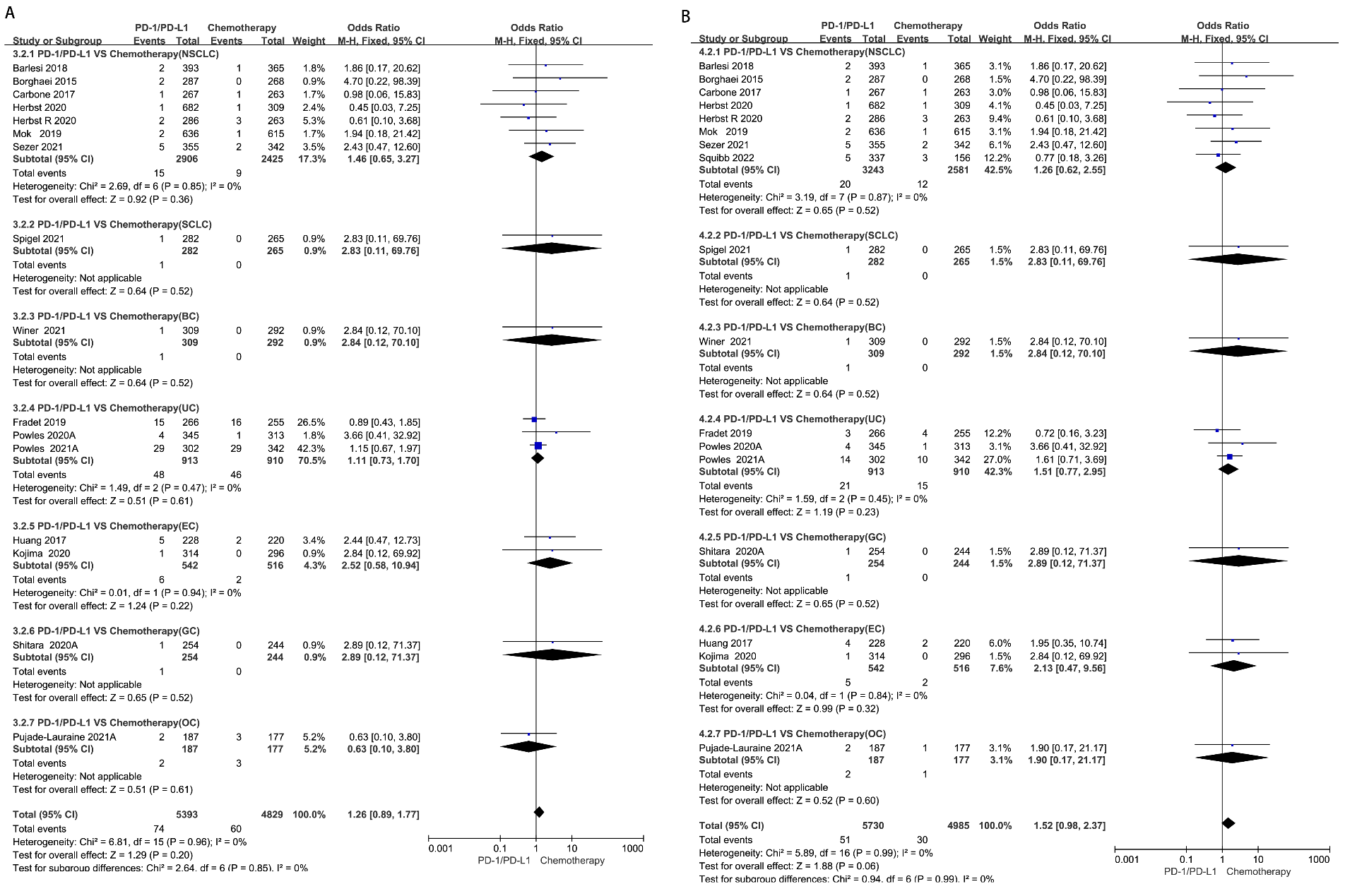


**Supplementary Figure 1.** Forest plots of the risk of all-grade and grade 3-5 cardiotoxicity calculated by the fixed effect model. **(A)** The risk of all-grade cardiotoxicity in PD-1/PD-L1 inhibitors VS Chemotherapy group (subgroup analysis was performed based on tumor types). **(B)** The risk of grade 3-5 cardiotoxicity in PD-1/PD-L1 inhibitors VS Chemotherapy group (subgroup analysis was performed based on tumor types).


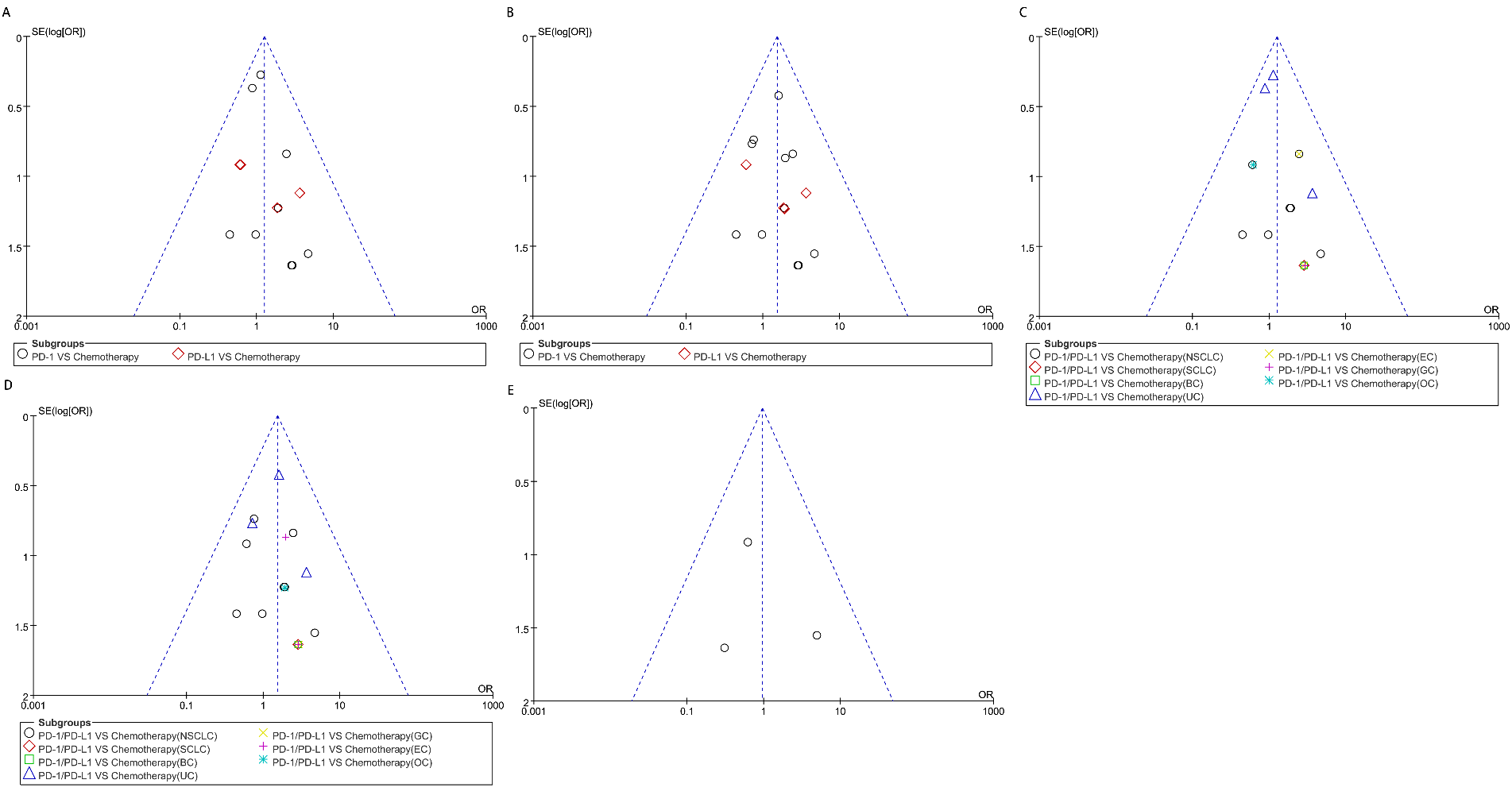


**Supplementary Figure 2.** Funnel plots of the risk of all-grade and grade 3-5 cardiotoxicity calculated by the fixed effect model. **(A)** The risk of all-grade cardiotoxicity in PD-1/PD-L1 inhibitors VS Chemotherapy group. **(B)** The risk of grade 3-5 cardiotoxicity in PD-1/PD-L1 inhibitors VS Chemotherapy group. **(C)** The risk of all-grade cardiotoxicity in PD-1/PD-L1 inhibitors VS Chemotherapy group (subgroup analysis was performed based on tumor types). **(D)** The risk of grade 3-5 cardiotoxicity in PD-1/PD-L1 inhibitors VS Chemotherapy group (subgroup analysis was performed based on tumor types). **(E)** The risk of all grade cardiotoxicity in PD-1/PD-L1 inhibitors+CTLA-4 inhibitors VS Chemotherapy group.


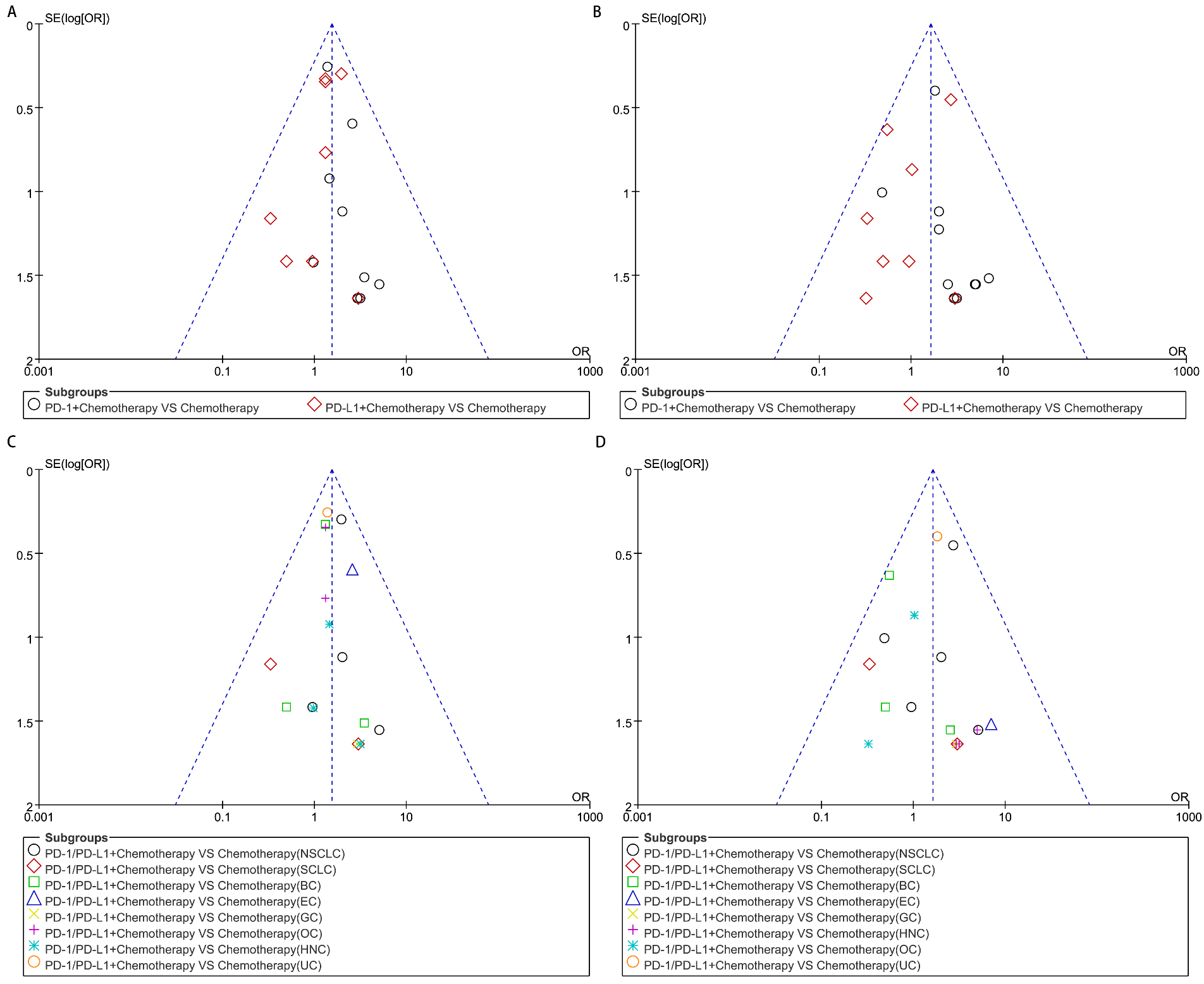


**Supplementary Figure 3.** Funnel plots of the risk of all-grade and grade 3-5 cardiotoxicity calculated by the fixed effect model. **(A)** The risk of all-grade cardiotoxicity in PD-1/PD-L1 inhibitors +Chemotherapy VS Chemotherapy group. **(B)** The risk of grade 3-5 cardiotoxicity in PD-1/PD-L1 inhibitors +Chemotherapy VS Chemotherapy group. **(C)** The risk of all-grade cardiotoxicity in PD-1/PD-L1 inhibitors + Chemotherapy VS Chemotherapy group (subgroup analysis was performed based on tumor types). **(D)** The risk of grade 3-5 cardiotoxicity in PD-1/PD-L1 inhibitors + Chemotherapy VS Chemotherapy group (subgroup analysis was performed based on tumor types).
